# Rupture traumatique du tendon tibial postérieur survenue lors d'une fracture fermée de la cheville: à propos d'un cas

**DOI:** 10.11604/pamj.2015.22.371.8427

**Published:** 2015-12-16

**Authors:** Mohamed Amine Karabila, Mohamed Azouz, Younes Mhamdi, Ismail Hmouri, Mohamed Kharmaz, Ahmed Bardouni, Abdou Lahlou, Mustapha Mahfoud, Mohamed Saleh Berrada

**Affiliations:** 1 Service de Chirurgie Orthopédique et de Traumatologie, CHU Ibn Sina, Rabat, Maroc

**Keywords:** Rupture, cheville, fracture, Rupture, ankle, fracture

## Abstract

Nous rapportons le cas d'une rupture post-traumatique du tendon tibial postérieur survenue lors d'une fracture bimalléolaire de la cheville. Le diagnostic a été posé lors de l'intervention chirurgicale. La réparation du tendon, non dégénératif, a été réalisée en même temps que l'ostéosynthèse. Bien que rare, cette possibilité de lésion tendineuse lors des fractures de la cheville ne doit pas êtreoubliée. Des douleurs résiduelles, un déficit de l'inversion active du pied, une modification de l'arche médiane du pied et à terme une évolution vers un pied plat valgus doivent faire évoquer rétrospectivement le diagnostic.

## Introduction

L'association d'une rupture du tendon du muscle tibial postérieur et d'une fracture de la cheville est rare. Dans la littérature anglo-saxonne, seuls 15 cas ont été publiées entre 1980 et 2000. Les lésions du tibial postérieur étaient survenues lors des fractures trimalléolaires, bimalléolaires, médiales isolées ou d’équivalents bimalléolaires [1–4]. Nous avons découvert fortuitement cette rupture du tendon lors de l'ostéosynthèse de la malléole médiale.

## Patient et observation

**Figure 1:**
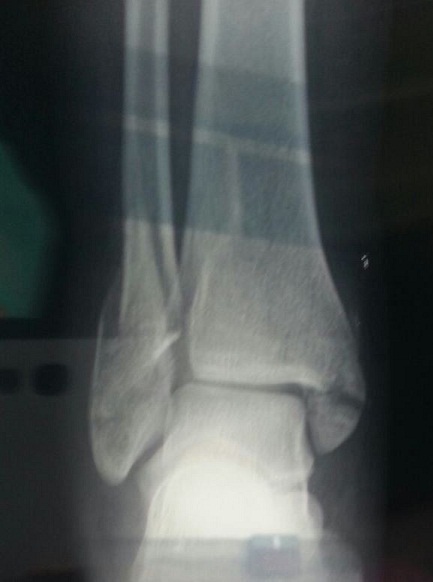
Radiographie de la cheville gauche montrant la fracture bimalléolaire

**Figure 2:**
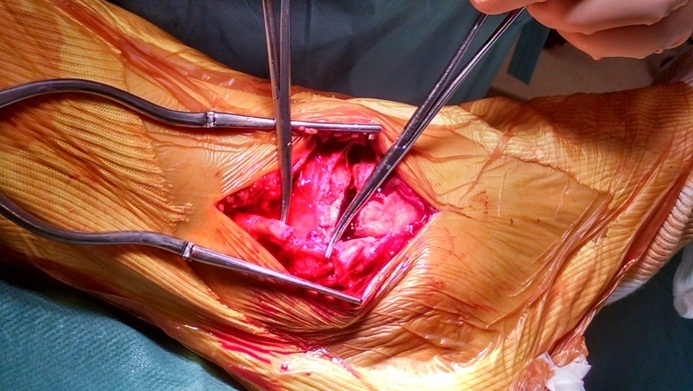
Vue per-opératoire montrant la rupture du tendon tibial postérieur

**Figure 3:**
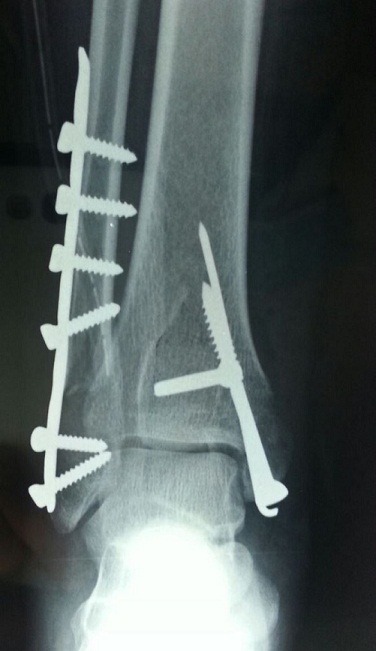
Radiographie de face cheville gauche montrant le contrôle post opératoire

M A, 36 ans, sans antécédent particulier notamment de tendinopathie, victime d'un accident de sport lors d'un match de handball suite à une chute sur le pieden éversionoccasionnant un traumatisme fermé de la chevillegauche. Le bilan radiologique standard a objectivé une fracture bimalléolaireinter-ligamentaire sans diastasis tibio-fibulaire (Figure 1). Lors de l'ostéosynthèse, nous avons commencé par la malléole externe qui était stabilisée par une plaque vissée et lors de l'abord chirurgical de la malléole médiale on constatait une rupture complète du tendon du muscle tibial postérieur à 1 cm au-dessus de la fracture;les extrémités de ce tendon étaient nettes (Figure 2). Après vissage de la malléoleinterne (Figure 3), le tendon a été réparé par un point en cadre par Vicryl 2/0 associé à un surjet au monofilament 4/0. Une botteplâtréeétait mise en place pour 6 semaines. Les suites opératoires étaient simples. La consolidation était complète à 3 mois. Le résultat fonctionnel était satisfaisant après 6 mois avec une mobilité de la cheville presque normale: flexion dorsale à 15^°^, une flexion plantaire à 20^°^ et le testing musculaire du tendon était fonctionnel.

## Discussion

Le tendon du muscle tibial postérieur joue un rôle majeur dans la statique et la dynamique du pied en assurant la stabilisation de l'arrière pied et de la voûte plantaire lors de la marche. Il s'agit d'un puissant tendon, antagoniste des tensons fibulaires, il est inverseur du pied. Sa situation anatomique rétro-malléolaire le rend peu mobile car le rétinaculum des fléchisseurs le plaque contre la malléole médiale. Il est plus médial des tendons cheminant en arrière de la malléole médiale et apparaît le plus prédisposé à la rupture lors des traumatismes de la cheville par éversion forcée. Il présente, d'après Frey et al. [5], une zone vasculaire lors de son passage en dessous et en arrière de la malléole médiale. Généralement, la rupture tendineuse survient sur un tendon pathologique: ténosynovites rhumatoïdes ou non spécifiques ainsi que des dégénérescences tendineuses [6, 7]. L'inspection préopératoire a montré dans notre cas un tendon sain. Le mécanisme lésionnel admis est une éversion brutale du pied avec tension maximale de la loge musculaire postérieure entraînant un étirement tendineux majeur, puis une rupture au niveau ou plus souvent au-dessus du trait de fracture. Le diagnostic clinique de rupture du tendon tibial postérieur est difficile a évoqué avant l'intervention. La douleur associée à la fracture, associée à un œdème des parties molles constituent une gêne à l'examen clinique et un obstacle au diagnostic. Seule une échographie ou une IRM permettraient de faire le diagnostic préopératoire, mais l'indication de ces examens est disproportionnée en hagard de la rareté des cas et de la fréquence des fractures de la cheville. La réduction et la stabilisation chirurgicale d'une fracture de la malléole médiale nécessitent la visualisation de la réduction non seulement en avant mais aussi en arrière pour ne pas méconnaître en peropératoire une lésion du tendon tibial postérieur et éliminer toute rotation du fragment. Une interposition inaperçue du tendon entre les fragments d'une fracture de la malléole médiale pourra conduire à une pseudarthrose lors d'un traitement orthopédique [8].

## Conclusion

Bien que rare, la rupture du tendon du tibial postérieur lors des fractures de la cheville pourrait être soupçonnée dans les situations suivantes: traumatisme initial violant, mécanisme en éversion, luxation associée, un trait de fracture malléolaire médiale bas situé. Dans ces cas, la synthèse en percutané apparait contre-indiquée.
